# Supplementary material for: Factors influencing place of delivery in Ethiopia: Linking individual, household, and health facility-level data
Source: PLOS Glob Public Health. 2022 Sep 1;2(9):e0000535. doi: 10.1371/journal.pgph.0000535 (PMC10021680; doi:10.1371/journal.pgph.0000535)
Supplement: S1 Table — (DOCX) [file pgph.0000535.s001.docx]

**Supporting information**

**S1 Table. Description of indicators used to measure facility readiness for delivery service**

| **Domains** | **Indicators/ items included** |
| --- | --- |
| **Equipment, supplies, and facilities/ amenities** | Sterile cord ties and scissors/blades; Hand washing facility; Infant scale; Obstetric forceps and/or electrical vacuum extractor; D&C kit and/or MVA; Suction apparatus; Newborn masks (size 0 and size 1); Resuscitation table; Electricity and/or back up energy source; Privacy of delivery room; Newborn corner; Sterilization equipment; Functional incubator; Oxygen supply; and Pulse oximeter |
| **Medicines and commodities** | Chlorohexidine gel; Injectable vitamin K; Tetracycline; BCG vaccine; Magnesium sulphate; Calcium gluconate; At least one antihypertensive (hydralazine, nifedipine, or methyldopa); Injectable diazepam, Injectable oxytocin; Misoprostol; Injectable ergometrine; Injectable ampicillin; Injectable gentamicin; Injectable metronidazole; Dexamethasone; Nevirapine (NVP); and Benzathine benzylpenicillin |
| **Staffing and systems to support quality** | The functional mechanism for reporting data on maternal deaths; Conducts maternal death reviews; Produces monthly reports and receives feedback that includes recommendations for action; Skilled birth attendant present or on-call 24h; Ratio of skilled health personnel to delivery volume meets; Has a performance monitoring team that meets at least quarterly; Access to a functional ambulance/car on-site for emergency transportation; Printed referral form observed; Functional system for recording and sharing outcomes of cases referred in and out |
| **Performance of signal functions** | Provide or perform: parenteral anticonvulsants; parenteral uterotonics; manual removal of placenta; instrument/ assisted deliveries; parenteral antibiotics; neonatal resuscitation; and antenatal corticosteroids in past 3 months |
